# Cafeteria Diet-Induced Obesity Worsens Experimental CKD

**DOI:** 10.3390/nu15153331

**Published:** 2023-07-26

**Authors:** Jonas Laget, Irene Cortijo, Juliana H. Boukhaled, Karen Muyor, Flore Duranton, Bernard Jover, Fabrice Raynaud, Anne-Dominique Lajoix, Àngel Argilés, Nathalie Gayrard

**Affiliations:** 1 RD-Néphrologie, 34090 Montpellier, France; jonas.laget@umontpellier.fr (J.L.); irene.cortijo-tejero@umontpellier.fr (I.C.); boukhaled@rd-n.org (J.H.B.); karenmuyor@gmail.com (K.M.); duranton@rd-n.org (F.D.); crecher34@gmail.com (B.J.); argiles@rd-n.org (À.A.); 2 PhyMedExp, INSERM, CNRS, Université de Montpellier, 34090 Montpellier, France; fabrice.raynaud1@umontpellier.fr; 3 Biocommunication in Cardio-Metabolism (BC2M), University of Montpellier, 34090 Montpellier, France; anne-dominique.lajoix@umontpellier.fr

**Keywords:** chronic kidney disease, obesity, adipokine, fibrosis, collagen, macrophage, inflammation

## Abstract

Obesity is a significant risk factor for chronic kidney disease (CKD). This study aimed to evaluate the impact of obesity on the development of kidney fibrosis in a model of cafeteria diet rats undergoing 5/6th nephrectomy (SNx). Collagen 1, 3, and 4 expression, adipocyte size, macrophage number, and the expression of 30 adipokines were determined. Collagen 1 expression in kidney tissue was increased in Standard-SNx and Cafeteria-SNx (7.1 ± 0.6% and 8.9 ± 0.9 tissue area, respectively). Renal expression of collagen 3 and 4 was significantly increased (*p* < 0.05) in Cafeteria-SNx (8.6 ± 1.5 and 10.9 ± 1.9% tissue area, respectively) compared to Cafeteria (5.2 ± 0.5 and 6.3 ± 0.6% tissue area, respectively). Adipocyte size in eWAT was significantly increased by the cafeteria diet. In Cafeteria-SNx, we observed a significant increase in macrophage number in the kidney (*p* = 0.01) and a consistent tendency in eWAT. The adipokine level was higher in the Cafeteria groups. Interleukin 11, dipeptidyl peptidase 4, and serpin 1 were increased in Cafeteria-SNx. In the kidney, collagen 3 and 4 expressions and the number of macrophages were increased in Cafeteria-SNx, suggesting an exacerbation by preexisting obesity of CKD-induced renal inflammation and fibrosis. IL11, DPP4, and serpin 1 can act directly on fibrosis and participate in the observed worsening CKD.

## 1. Introduction

The prevalence of obesity has been growing worldwide due to the combination of sedentary lifestyles and overconsumption of diets rich in fat, salt, and sugar [1], and 11 million deaths were attributed to dietary risk factors in 2017 [2]. Moreover, the prevalence of obesity worldwide has risen among children and adolescents (5–19 years) in the last four decades, from around 11 million in 1975 to more than 120 million in 2016 [3]. In the last report from the Centers for Disease Control and Prevention (CDC), the prevalence of obesity among young people (2–19 years) was 19.7% in the USA [4]. For many chronic diseases, excess weight is a leading risk factor linked to shortened life expectancy [5], and a high body mass index in adolescence leads to an increased risk of death from cardiovascular disease [6]. Obesity can be associated with type 2 diabetes, arterial hypertension, cardiovascular diseases, and other metabolic disorders. Such metabolic disorders are important risk factors for chronic kidney disease (CKD), highlighting the tight relationship between obesity and CKD [7]. In a renal biopsy-based clinicopathologic study, the incidence of obesity-related glomerulopathy increased from 0.2% in 1986–1990 to 2.0% in 1996–2000 [8]. In 2006, obesity was responsible for 16% of new CKD cases in men and 11% in women according to a Swedish study [9]. Interestingly, a higher risk of developing CKD is also present in metabolically healthy obese patients compared to normal-weight individuals [10]. This supports the hypothesis of the direct effect of obesity on renal health, for which alternative pathways independent from arterial hypertension or type 2 diabetes effects should be considered [9].

Obesity has been shown to induce low-grade chronic inflammation [11,12,13]. More specifically, obesity causes “meta-inflammation”, where specific inflammatory metabolic factors replace classical inflammatory molecules [12]. In adipose tissue, obesity triggers adipocyte hypertrophy, macrophage recruitment, and adipokine production that participate in the development of chronic inflammation and insulin resistance (IR) [13,14] and aggravate CKD [15]. Obesity induces hemodynamic and morphologic changes in the kidney that could result in reduced (renal function) GFR [16,17]. IR is associated with the level of renal interstitial fibrosis in non-diabetes CKD patients [18], and more generally with CKD progression [19]. In addition, uremic toxins and the inflammation triggered by CKD can facilitate fat accumulation and activate immune cells in the adipose tissue to a more proinflammatory phenotype leading to obesity worsening [20].

Overall, obesity promotes the development and progression of renal disease. Still, the relationship between obesity-related pro-inflammatory adipose tissue and renal tissue fibrosis progression in CKD remains to be elucidated.

Our study aimed to evaluate the impact of pre-existing obesity on the development of kidney fibrosis in a 5/6 nephrectomy rat model fed a «cafeteria diet» [21]. In particular, we report a rise in inflammatory mediators, including macrophages in renal and adipose tissues and adipokines secreted by adipose tissue, validating the theory that meta-inflammation worsens CKD.

## 2. Materials and Methods

### 2.1. Animals

All present animal experiments complied with European and French laws (Agreement D34-172-25 and 1562–18,348) and conformed to the Guide for the Care and Use of Laboratory Animals published by the National Institutes of Health [22].

**Figure 1:**
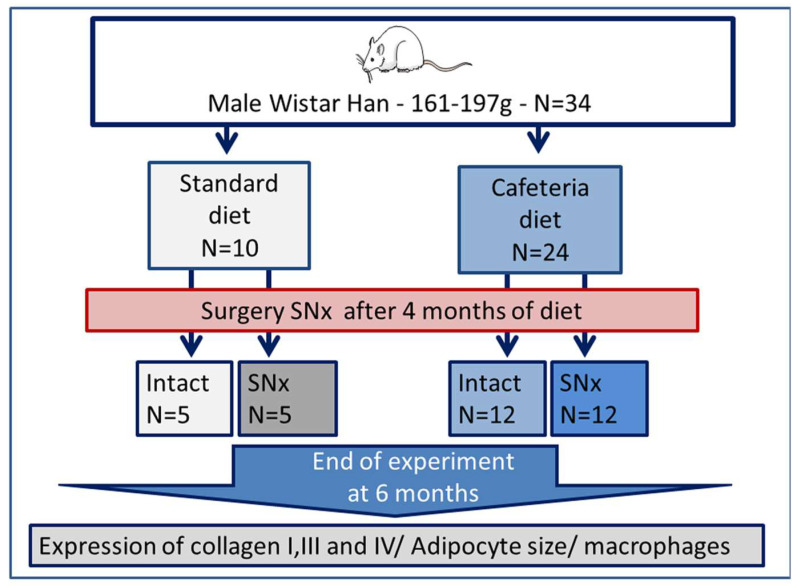
Study design of the animal experiment. SNx: subtotal nephrectomy.

All animals were housed from April to October 2017 with free access to their respective diets and water and maintained on a light–dark cycle. The impact of obesity on the development of kidney fibrosis was studied using a previously described model of renal mass reduction and cardiac remodeling assessment on 34 rats [23]. Six-week-old male Wistar rats (Charles River Laboratories) were utilized, 24 were fed a cafeteria diet and 10 a standard diet (see Supplementary Figure S1, A04, SAFE Diets) over a period of 6 months (Figure 1). The cafeteria diet was adapted from Sampey et al. [21] and consisted of crackers, cereals, cookies, and processed meat (Supplementary Table S1) added to 5 g/day/rat standard diet. The items used in the cafeteria diet were chosen to be representative of the highly palatable, unhealthy, energy-dense food found in the Western diet and were rich in fat, sugar, and salt while poor in dietary fiber. Half of the rats that were fed either the standard or cafeteria diet underwent subtotal nephrectomy (SNx) at 4 months old. Two branches of the left renal artery were ligated and the right kidney was removed in a one-step procedure [24]. The study was therefore conducted in four groups of rats: standard diet (Standard), cafeteria diet (Cafeteria), standard diet with SNx (Standard-SNx), and cafeteria diet rat with SNx (Cafeteria-SNx). Two months after the SNx surgery, the rats were sacrificed through anesthesia (isoflurane 2%), and blood was sampled and centrifuged at 1000× *g* for 10 min at 4 °C, and plasma was collected. Kidney and epididymal white adipose tissues (eWAT) were taken and preserved in formalin and snap-frozen in liquid nitrogen for histological analysis and for protein investigation such as Western blot, adipokine measurement, and immunostaining. All the collected biological samples except the formalin samples were stored at −80 °C, until further analysis. Rat body weight was measured weekly, eWAT was weighed, and tibia length was measured at the end of protocol.

### 2.2. Blood Parameters

Plasma creatinine was determined on a COBAS automated analyzer (Roche Diagnostics, Meylan, France). Blood was collected from the tail artery after 5 h of fasting three days before the end of protocol for glucose and insulin determination. The blood glucose meter Freestyle Optium Neo was used to measure the glucose and insulin was quantified by an rat insulin ELISA kit (Mercodia, Uppsala, Sweden). The homeostasis model assessment of insulin resistance (HOMA-IR) was calculated using the following equation: HOMA-IR = (fasting blood glucose in mg/dL × fasting plasma insulin in mU/L)/2430 [25].

### 2.3. Histology

Formalin eWAT samples were embedded in paraffin and 5 µm sections made with a microtome (HistoCore Multicut, Leica Biosystems, Nanterre, France) were placed on glass slides for staining. To evaluate adipocyte size, the eWAT tissues were stained by hematoxylin QS (Vector Laboratories, Newark, CA, USA). The sections were mounted in mounting medium, Entelan (Merck, Saint-Quentin-Fallavier Cedex, France) and examined under a light microscope (Nikon Eclipse TE300). For each sample, quantification was performed with Image J 1.53c software on 10 photos taken at 200-fold magnification. Adipocyte size and distribution were calculated according to Parlee’s article [26].

### 2.4. Immunochemistry

Immunohistochemistry was performed in 5 µm kidney cryosections (from snap-frozen samples) made with a cryostat (CM1850, Leica, Wetzlar, Germany) and formalin eWAT samples. Primary antibodies, anti-collagen 1 (1:200,ABCAM, Paris, France), anti-collagen3 (1:200, Abcam, Singapore), anti-collagen 4 (1:200, Abcam), and anti-CD68 (1:200, Biorad, Marnes la Coquette, France) were incubated overnight at 4 °C. Revelation and staining quantification were performed as previously described [27] and according to supplier’s instructions using Universal Vectastain ABC kit and ImmPACT AEC 130 (Vector Laboratories). In eWAT, the number of macrophages was counted per microscopic field at 200× magnification.

### 2.5. Adipokine Determination

Following manufacturer’s instructions, 200 mg of eWAT was processed for adipokine evaluation using a Rat Adipokine Array Kit (#ARY016, R&D Systems, Minneapolis, MN, USA). Briefly, snap-frozen tissue was placed in 2 ml screw cap vials filled with 1.0 mm dia Zirconia/Silica lysis beads (#11079110z, BioSpec Products, Bartlesville, OK, USA) and PBS1X adjusted with Protease/Phosphatase Inhibitor Cocktail (#5872S, Cell Signaling, Danvers, MA, USA) at 1× working concentration. The samples were homogenized using a FastPrep-24 homogenizer (MP Biomedicals, Illkirch, France) with 2 cycles of 30 s at 5 m/s with cooling on ice for 5 min between cycles. After homogenization, triton X-100 was added to a final concentration of 1%, and samples were frozen at −80 °C. The samples were thawed and centrifuged at 10,000× *g* for 5 min to remove cellular debris, and the supernatant was collected. Quantitation of sample protein concentration was determined by the BCA method (#23225, Thermo-Scientific, Courtaboeuf, France), and 250 µg of protein was deposited on the adipokine array membranes. Membranes were exposed for 2 min. The expressions of 30 adipokines were determined. The pixel density of each spot was quantified with Image Studio™ Lite 3.1 software (LI-COR Biotechnology, Lincoln, NE, USA). The signal intensity was calculated as the average pixel density from duplicate spots from which the membrane average background signal was subtracted. The assays were carried out in the tissues of the four groups of rats randomized in batches of 4 membranes. Batch-specific factors were applied to match all batches’ signal intensities (i.e., sum of all signals from all samples for each batch).

### 2.6. Statistical Analysis

Statistical analyses were performed with SAS v9.4 (SAS Institute, Cary, NC, USA). Continuous variables were checked for normality based on data distribution, and normalization by log transformation was performed when appropriate. We tested differences between conditions by two-way ANOVAs using the diet (Cafeteria or Standard), the surgery (SNx or Sham), and their interaction (Cafeteria × SNx) as explanatory factors, followed by Tukey’s post hoc tests. *p*-values < 0.05 were considered statistically significant. Unless stated otherwise, summary data are represented as mean ± standard error of the mean.

## 3. Results

### 3.1. Influence of Cafeteria Diet and SNx on Kidney Function and Insulin Resistance

As previously reported [21] and presented in Table 1, SNx rats had increased serum creatinine levels, which were even higher in the Cafeteria-SNx group (70.5 ± 10.3 µM) compared to Standard-SNx (54.0 ± 7.0 µM). Serum creatinine level was comparable in intact animals fed the cafeteria (31.7 ± 1.2 µM) or standard diet (34.2 ± 5.1 µM). The left kidney weight of Cafeteria-SNx was significantly higher (1833 ± 160 mg) than Cafeteria’s (1332 ± 34 mg). In rats fed the cafeteria diet, plasma insulin and HOMA-IR were significantly higher than in Standard animals, indicating diet-related insulin resistance. Whatever the diet, SNx surgery was associated with decreased plasma insulin and HOMA-IR. The glycemia was unchanged in the four groups (Table 1).

### 3.2. Effect of the Cafeteria Diet and SNX Surgery on Body Weight and Adipose Tissue

**Figure 2:**
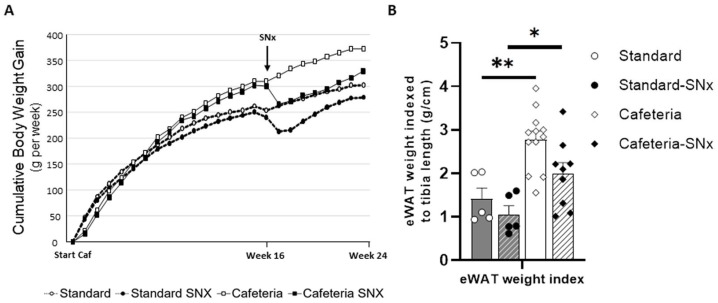
Body and epididymal white adipose tissue weights increase with cafeteria diet and reduce in 5/6 nephrectomized rats: (**A**) Evolution of rat body weight during the experiment; (**B**) Adiposity index (eWAT mass (g)/Tibia length (cm). Adiposity is higher in Cafeteria groups (** *p* < 0.001 between Standard and Cafet, and * *p* < 0.05 between Standard SNx and Cafet SNx).

Except at the onset of the enriched diet, the body weight gain of rats fed a cafeteria diet increased faster than in rats fed a standard diet (Figure 2A). SNx surgery resulted in a weight loss of about 29 g for 1 week. Then, weight increase restarted similarly in SNx rats on the standard or cafeteria diet with a cumulative gain of 38 ± 6 and 38 ± 4 g/6 weeks, respectively. At the end of the experimental time, the body weight of SNx rats remained lower than intact rats fed the corresponding diet.

The adiposity index of eWAT (Figure 2B) was increased by the cafeteria diet in sham (*p* < 0.001) and in SNx (*p* < 0.005). Concerning the body weight, it tended to decrease with SNx (both, Standard-Sham vs. Standard-SNx or Cafeteria-Sham vs. Cafeteria-SNx).

### 3.3. eWAT Examination: Adipocyte Sizes and Size Repartition

**Figure 3:**
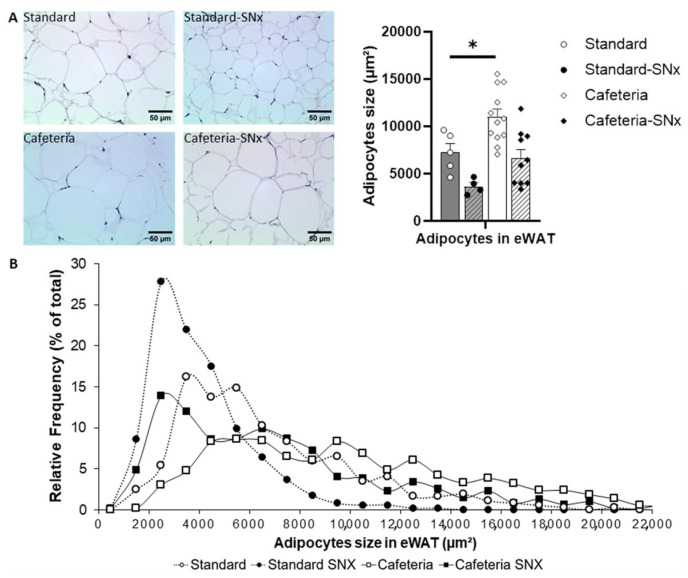
Adipocyte size and their relative frequency distributions in eWAT: (**A**) Adipocyte surface area measurements; (**B**) Relative frequency distributions of adipocyte surface areas (%). The adipocyte size of Cafeteria rats is significantly higher in Standard rats (* *p* = 0.05).

As shown in Figure 3A, adipocyte size increased significantly with the cafeteria diet (Cafeteria vs. Standard, respectively, 11,006 ± 817 µm^2^ and 7261 ± 938 µm^2^, *p* = 0.05), and there was a trend to increase in Cafeteria-SNx rats compared with Standard-SNx rats (6628 ± 914 µm^2^ and 3659 ± 411 µm^2^, respectively). In the Standard group, most adipocytes were between 3000 and 6000 µm^2^, whereas, in the Cafeteria group, adipocyte size was predominantly between 4000 and 10,000 µm^2^, reflecting a shift toward larger adipocytes (Figure 3B). In SNx rats (Standard-SNx and Cafeteria-SNx), a majority of observed adipocytes had a small size, around 2500 µm^2^. Similarly, in the Standard-SNx and Cafeteria-SNx groups, fewer large adipocytes (size greater than 5000 µm^2^ for Standard and greater than 9000 µm^2^ for Cafeteria) were observed compared to the respective sham groups (Figure 3B). SNx tended to reduce adipocyte size in rats fed the standard or cafeteria diet (Figure 3A,B).

### 3.4. Kidney Fibrosis

**Figure 4:**
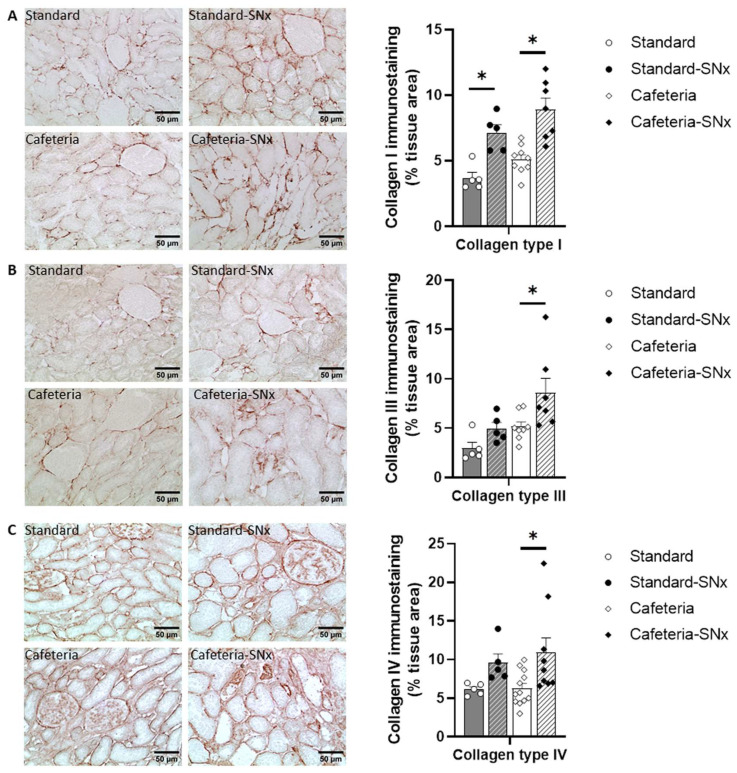
Evaluation of collagen 1, 3, and 4 abundance by immunohistochemistry in kidney sections: (**A**) Collagen 1; (**B**) Collagen 3, and (**C**) Collagen 4 quantifications. Collagen 1 is higher in SNx group (* *p* < 0.05 between Standard and Standard SNx, and between Cafet and Cafet SNx). Collagen 3 and 4 are higher in Cafeteria SNx compared to Cafeteria group (* *p* < 0.05 both).

Kidney fibrosis was evaluated by the quantification of the amount of collagen 1, 3, and 4, which are the most abundant collagens found in the kidney (Figure 4). Collagen 1 significantly increased in Standard-SNx and Cafeteria-SNx (Figure 4A, 7.1 ± 0.6% and 8.9 ± 0.9% tissue area, respectively) compared to their corresponding groups (3.7 ± 04% and 5.1 ± 0.4% tissue area, respectively). Renal expression of collagen 3 (Figure 4B) and collagen 4 (Figure 4C) was significantly increased in Cafeteria-SNx (8.6 ± 1.5 and 10.9 ± 1.9% tissue area, respectively) compared to Cafeteria rats (5.2 ± 0.5 and 6.3 ± 0.6% tissue area, respectively).

### 3.5. Inflammation in Kidney and eWAT

**Figure 5:**
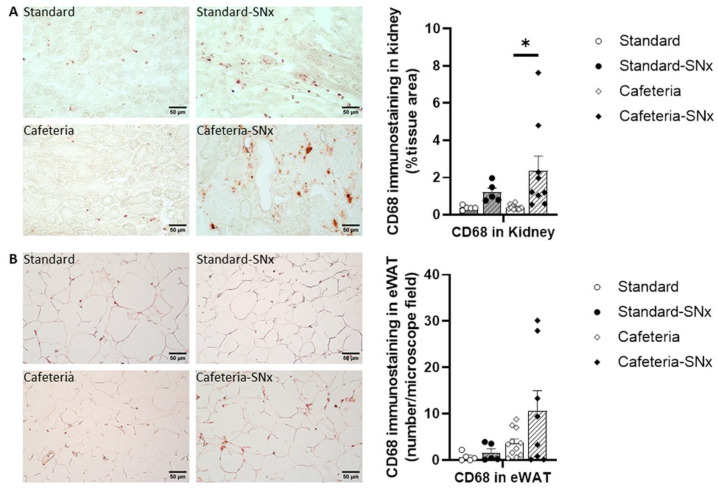
Macrophage number in kidney and adipose tissue: (**A**) Kidney and (**B**) Adipose tissue. The CD68 immunostaining in kidney tissue is higher in Cafeteria SNx compared to Cafeteria (* *p* < 0.05) and the macrophage numbers in adipose tissue tend to increase in Cafeteria SNx compared to Cafeteria.

**Figure 6:**
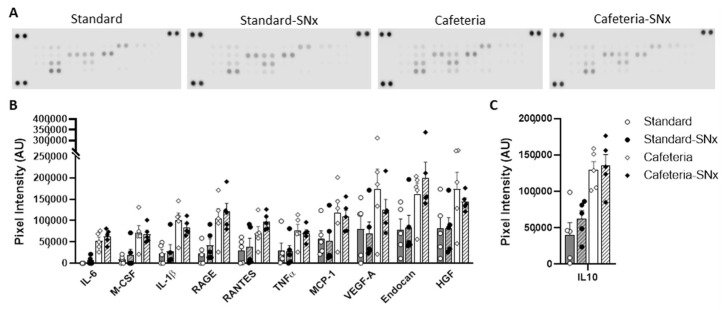
Adipokine investigation in epididymal white adipose tissue: (**A**) Representative picture of the adipokine array membranes for each group of rats. (**B**) Levels of ten pro-inflammatory cytokines (RANTES, endocan, MCP1, RAGE, interleukin 6, interleukin 1β, MCSF, HGF, tumor necrosis factor-alpha, and VEGFA) and (**C**) levels of IL10 measured by mean pixel density.

The presence of macrophages was investigated in the kidney and eWAT by immunostaining with the CD68 antibody (Figure 5). In the Cafeteria-SNx group, CD68 immunostaining showed a significant increase in the macrophage number in the kidney (Figure 5A, 2.36 ± 0.79% tissue area) compared to Cafeteria (0.39 ± 0.04% tissue area). A similar trend was observed in eWAT (Figure 5B) (10.6 ± 4.3 vs. 3.7 ± 0.9 macrophages per field). Regarding the inflammation status of eWAT, the level of 30 adipokines was assessed in eWAT using a cytokine antibody array (Figure 6A and Table 2). Preadipocyte factor 1 (Pref-1) [28], fibroblast growth factor 21 (FGF-21) [29], leukemia inhibitory factor (LIF) [30], angiopoietin-like 3 [31], and interleukin 11 [32,33], all implicated in lipid metabolism or adipogenesis, were significantly increased in both Cafeteria groups compared to their corresponding Standard groups (Table 2). Interestingly, interleukin 11 (Il11) has pro-fibrotic properties in the kidney [34]. The dipeptidyl peptidase 4 (DPP4), which plays a major role in glucose metabolism and may be implicated in fibrosis [35,36], was also increased in Cafeteria-SNx rats compared to Standard-SNx (Table 2). Leptin was decreased in the SNx groups, which could be linked to weight loss as previously observed in rodent models subjected to caloric restriction (Table 2) [37,38].

The level of ten pro-inflammatory cytokines (RANTES, endocan, MCP1, RAGE, interleukin 6, interleukin 1β, MCSF, HGF, tumor necrosis factor-alpha, and VEGFA) was significantly higher in the Cafeteria groups compared to the Standard groups and the first seven adipokines mentioned were increased in the Cafeteria-SNx group compared to the Standard-SNx group (Figure 6B, Table 2). Interleukin 10, classified as an anti-inflammatory cytokine, followed the same variation as that of the seven inflammatory cytokines (Figure 6C, Table 2). The intercellular adhesion molecule ICAM1 was significantly reduced in the SNx group (Table 2).

Among the adipokines related to the insulin-like growth factor, IGFII and IGFBPI were increased in the Cafeteria groups and only IGFBPI significantly increased in the Cafeteria-SNx group compared to the Standard-SNx group (Table 2). IGFBP6 decreased in the SNx groups regardless of diet type (Table 2).

The tissue inhibitor of metalloproteinase TIMP1 increased significantly in the cafeteria groups (Table 2). In Cafeteria-SNx rats compared to the Standard-SNx, the serine proteinase inhibitor serpin E1, known as pro-fibrotic in the kidney [39], was significantly higher (Table 2).

Two of the most expressed adipokines, resistin and lipocalin2, were not modified by the cafeteria diet and/or SNx (Table 2).

## 4. Discussion

In the present study, we induced CKD by SNx surgery in rats with pre-existing obesity to evaluate the influence of overweight on CKD progression. The cafeteria diet led to body weight gain and adipose tissue hypertrophy (increase), which was partially blunted in SNx rats. Adipocyte size followed the same pattern, i.e., an increase with the cafeteria diet was partially blunted in SNx rats. This was consistent with the fact that surgery-induced CKD was typically accompanied by loss of body weight [40,41,42]. Yet, the effect of the cafeteria diet on adipocyte size was similar in intact and SNx rats when compared to their corresponding standard groups (approximately 51% and 81%, respectively). Regarding renal function reduction, obese SNx rats had higher plasma creatinine levels compared to SNx rats. This was consistent with various clinical studies that have shown an increased risk of CKD development and progression in diabetic or non-diabetic obese patients [43,44,45].

The assessment of collagens 1, 3, and 4 in renal tissue revealed increased amounts of collagen 1 in SNx rats in obese and standard diet-fed animals. The amount of collagen 3 and 4 in the kidney was higher in SNx rats fed the cafeteria diet, supporting the link between obesity and renal fibrosis [16]. Macrophage infiltration was also increased in the kidney of Cafeteria-SNx rats, showing an exacerbation of inflammation in overweight, which is known to participate in the progression of CKD [46]. In the present work, the obesity was moderate compared to other studies reporting much bigger body weight gains [47,48,49]. The obese rats were insulin-resistant and had higher plasma insulin levels and HOMA-IR, but were not diabetic. This was close to the concept of metabolically healthy obesity (MHO), which is defined as the absence of type 2 diabetes mellitus, dyslipidemia, arterial hypertension, and atherosclerotic cardiovascular disease in patients with obesity or overweight status [10]. In obese rats, we observed an alteration of adipose tissue that led to the secretion of pro-inflammatory cytokines and a decrease in insulin sensitivity, which were potential causes for the faster CKD progression observed.

In SNx obese rats, we observed adipocyte hypertrophy, macrophage recruitment, and modified adipokine secretion; the majority of adipokines being at a higher level. This was not surprising since the dysregulation of adipokines secretion is a frequent feature in obesity and metabolic diseases [50,51]. Interestingly, 7 out of the 10 proinflammatory cytokines were increased in eWAT in obese rats including SNx compared to the respective controls. Based on previous evidence of inter-organ crosstalk [49,52,53], eWAT inflammation was likely to affect the inflammatory status of kidneys, and may be partly responsible for the observed infiltration of macrophages in kidney tissue [46]. Among the five cytokines related to lipid metabolism and adipogenesis that increased in SNx obese rats, the presence of IL11 should be noted. IL11 is an important downstream regulator of TGF-β and has been recently described as a pro-fibrotic factor in fibroblasts as well as in a mouse model of acute kidney injury [34,54,55]. In our SNx obese model, this cytokine could be linked to the worsened CKD and renal fibrosis, as IL11 release in the circulation by adipose tissue can stimulate renal epithelial cell mesenchymal transition and dysfunction, as has been previously demonstrated in cultured cells [34,56,57].

DPP4, which was found to be increased in the SNx obese rats, is implicated in glucose metabolism by interaction with the incretin system that regulates glycemia through the amplification of insulin secretion. Previous studies have shown that DPP4 is secreted from adipose tissue and its expression was correlated with adipocyte size and adipose tissue inflammation [58,59]. DPP4 inhibitors have a protective role in models of hepatic fibrosis [35,36] and renal fibrosis [36], which implies that DPP4 is pro-fibrotic and promotes CKD in SNx obese rats.

Finally, serpin E1, also known as plasminogen activator inhibitor-1, was increased in obese SNx rats and may contribute to kidney fibrosis according to its pro-fibrotic properties in renal epithelial cells [39].

## 5. Conclusions

In summary, cafeteria diet-induced obesity exacerbated fibrosis (collagen 1 and 4 levels) and macrophage infiltration in the kidneys of SNx rats. In adipose tissue, our study has shown a dysregulation of adipokine production that may affect targeted tissues such as kidneys and lead to CKD progression. We identified three candidate adipokines that may act directly on renal fibrosis and be involved in the worsening of CKD by obesity: IL11, DPP4, and serpin 1. Further studies on these adipokines should aim to elucidate their mechanisms of action and role in the inter-organ crosstalk that participates in the observed worsening of CKD by obesity.

## Figures and Tables

**Table 1:** Plasma creatinine, glycemia, insulin resistance, and left kidney weight evaluation in the rats after standard or cafeteria diet and Sham or SNx surgery.

	Standard-Sham	Standard-SNx	Cafeteria-Sham	Cafeteria-SNx
Creatinine µmol/L	34.2 ± 5.1	54.0 ± 7.0 *	31.7 ± 1.2	70.5 ± 10.3 *
Glycemia mg/dL	90 ± 2	94 ± 2	95 ± 2	91 ± 3
Insulin mU/L	92 ± 17	66 ± 22	177 ± 13 †	112 ± 19 *†
HOMA-IR	3.3 ± 0.8	2.6 ± 1.0	6.9 ± 0.6 †	4.4 ± 0.7 *†
Left kidney Wt mg	1250 ± 34	1477 ± 82	1332 ± 34	1833 ± 160 *†

HOMA-IR: homeostatic model assessment for insulin resistance. Wt: weight. SNx: subtotal nephrectomy. * indicates *p* < 0.05 vs. sham fed the same diet indicating an “SNx effect”; † indicates *p* < 0.05 vs. Standard with the same renal surgery indicating a “diet effect”.

**Table 2:** Mean adipokine signal intensity in the different groups of rats (arbitrary unit).

	Mean Level ± Sem				*p*-Value		
	Standard-Sham	Standard-SNx	Cafeteria-Sham	Cafeteria-SNx	Surgery Effect (SNx vs. Sham)	Diet Effect (Cafeteria vs. Standard)	Diet Effect in SNx (Cafeteria-SNx vs. Standard-SNx)
**Pref1**	13 ± 6	27 ± 14	83 ± 13	128 ± 29	0.12	**<0.001**	**<0.01**
**FGF21**	30 ± 13	37 ± 6	126 ± 17	152 ± 22	0.28	**<0.001**	**<0.001**
**LIF**	62 ± 14	66 ± 20	131 ± 22	161 ± 10	0.33	**<0.001**	**<0.01**
**Angiopoietinlike3**	107 ± 28	86 ± 18	115 ± 15	173 ± 8	0.35	**0.02**	**0.02**
**IL11**	137 ± 22	59 ± 18	132 ± 12	185 ± 31	0.56	**0.01**	**<0.01**
							
**DPPIV**	350 ± 45	248 ± 23	356 ± 27	419 ± 13	0.52	**<0.01**	**<0.01**
							
**Leptin**	824 ± 126	392 ± 58	802 ± 87	583 ± 96	**<0.01**	0.39	**0.50**
							
**RANTES**	26 ± 11	33 ± 17	72 ± 15	105 ± 10	0.16	**<0.001**	**<0.01**
**Endocan**	74 ± 20	78 ± 16	166 ± 26	212 ± 35	0.33	**<0.001**	**<0.01**
**MCP1**	57 ± 16	47 ± 16	114 ± 19	115 ± 15	0.77	**<0.01**	**0.04**
**RAGE**	20 ± 10	40 ± 11	101 ± 15	127 ± 14	0.09	**<0.001**	**<0.01**
**IL6**	0 ± 0	5 ± 3	54 ± 9	70 ± 13	0.22	**<0.001**	**<0.001**
**IL1b**	19 ± 9	24 ± 10	100 ± 12	91 ± 12	0.83	**<0.001**	**<0.01**
**MCSF**	8 ± 3	15 ± 10	69 ± 10	73 ± 10	0.52	**<0.001**	**<0.01**
**HGF**	77 ± 25	75 ± 15	170 ± 28	155 ± 14	0.70	**<0.001**	0.07
**TNFa**	30 ± 17	26 ± 12	76 ± 11	76 ± 12	0.88	**<0.01**	0.07
**VEGFA**	74 ± 28	64 ± 17	163 ± 33	127 ± 12	0.35	**<0.01**	0.29
							
**IL10**	37 ± 16	68 ± 18	138 ± 14	150 ± 28	0.30	**<0.001**	**0.049**
**ICAM1**	915 ± 85	781 ± 49	1077 ± 73	793 ± 36	0.00	0.19	0.99
							
**IGFBP1**	51 ± 11	39 ± 11	94 ± 9	147 ± 31	0.28	**<0.001**	**<0.01**
**IGFBP2**	234 ± 75	97 ± 34	235 ± 36	243 ± 33	0.20	0.14	0.18
**IGFBP3**	1267 ± 159	989 ± 140	1115 ± 123	1368 ± 67	0.92	0.39	0.19
**IGFBP5**	950 ± 60	804 ± 32	875 ± 85	804 ± 78	0.13	0.59	0.99
**IGFBP6**	1716 ± 90	1420 ± 70	1784 ± 95	1439 ± 162	0.01	0.70	0.99
**IGFI**	312 ± 111	212 ± 30	273 ± 43	530 ± 203	0.52	0.26	0.27
**IGFII**	46 ± 16	67 ± 13	152 ± 19	162 ± 20	0.39	**<0.001**	**<0.01**
							
**TIMP1**	134 ± 31	211 ± 66	349 ± 82	452 ± 101	0.24	**<0.01**	0.14
**SerpinE1**	30 ± 9	39 ± 10	94 ± 12	131 ± 22	0.13	**<0.001**	**<0.01**
							
**Resistin**	1833 ± 131	1382 ± 183	1374 ± 93	1546 ± 100	0.30	0.28	0.82
**Lipocalin2**	1590 ± 563	1169 ± 233	1432 ± 368	2136 ± 418	0.74	0.34	0.38

Note: For each adipokine, we performed a two-way ANOVA using diet, surgery, and interaction diet × surgery as explanatory factors, followed by Tukey’s post hoc tests. Colors represent minimal (green), median (yellow) and maximum (red) signal intensities. *p*-values < 0.05 are in bold.

## Data Availability

The datasets generated and analyzed during the current study are available from the corresponding author on reasonable request.

## Supplementary Materials

The following supporting information can be downloaded at: https://www.mdpi.com/article/10.3390/nu15153331/s1, Figure S1: SAFE A04 scientific diet product data sheet (extract); Table S1: Composition of the items used in the Cafeteria diet group.
