# Supplementary material for: Cafeteria Diet-Induced Obesity Worsens Experimental CKD
Source: Nutrients. 2023 Jul 26;15(15):3331. doi: 10.3390/nu15153331 (PMC10421241; doi:10.3390/nu15153331)
Supplement: Supplementary file 1 [file nutrients-15-03331-s001.zip › nutrients-2509116-supplementary.pdf]

Cafeteria Diet-Induced Obesity Worsens Experimental CKD

Jonas Laget [1], Irene Cortijo [1], Juliana H. Boukhaled [1], Karen Muyor [1], Flore Duranton [1], Bernard Jover [1], Fabrice Raynaud [2], Anne-Dominique Lajoix [3], Àngel Argilés [1] and Nathalie Gayrard [1,*]

[1] RD-Néphrologie, 34090 Montpellier, France
[2] PHYMEDEXP, INSERM, CNRS, Université de Montpellier, 34090 Montpellier, France
[3] Biocommunication in Cardio-Metabolism (BC2M), University of Montpellier, 34090 Montpellier, France
* Correspondence: nathalie.gayrard@umontpellier.fr

Supplementary file

Table S1. Composition of the items used in the Cafeteria diet group

Figure S1. SAFE A04 scientific diet product data sheet (extract)

Table S1. Composition of the items used in the Cafeteria diet group.

	Energy (kcal/100g)	Fat (g/100g)	Saturated Fat (g/100g)	Carbohydrates (g/100g)	Sugars (g/100g)	Dietary Fiber (g/100g)	Proteins (g/100g)	Salt (g/100g)
Pound Cake (Chabrior)	431	24.4	15.9	46.6	27.3	< 0.5	5.7	1.26
Liege Wafles (Chabrior)	443	22.5	11.1	54.1	26.3	1.6	5.3	0.57
Butter biscuit (Chabrior)	515	26.8	18.6	62.2	23.5	1.7	5.4	1.13
Salami (Top Budget)	442	42	17	0.5	< 0.5	0	16.3	4
Bacon (Monique Ranou)	253	21	8.1	0.6	0.5	< 0.5	15.5	2.2
Gouda (Paturages)	358	28.6	19.1	< 0.5	< 0.5	< 0.5	23.4	2.02
Comté (Paturages)	413	34	23	0.8	< 0.5	< 0.5	26	0.8
Salt Chips (Lay's)	551	34	4.2	53	0.5	4.2	6.3	1.1
Peanut Chips (Vico)	486	24	3.2	52	1.4	5.5	13	1.5
Salt Chips "Fritelle" (Bénenuts)	439	15	1.2	69	1	2.5	7.3	2.4
Salt Crackers (LU)	479	19	1.9	67	7.1	2.4	8.4	1.7
Pringles Chips (Pringles)	534	31	6.6	56	1.4	3.5	5.9	1.1
Breakfast Bar with Nuts (LU)	461	22	4.2	61	18	3.7	6.6	0.6
White chocolat (Nestlé)	561	34.3	20.1	54	54	0	8.2	0.3
Marshmallows (Haribo)	326	0.5	0.1	80	64	0	2.6	0.02
Marzipan (Vahiné)	430	17	13	61	53	4.3	7.3	0

SAFE® A04

Ingredients

Barley, wheat, maize, soybean meal, wheat bran, hydrolyzed fish proteins, dicalcium phosphate, pre-mixture of minerals, calcium carbonate, pre-mixture of vitamins.

CENTESIMAL COMPOSITION

Cereals	84.1 %
Animal Proteins	4.0 %
Vegetal Proteins	8.0 %
Vitamins & Minerals	3.9 %

NUTRITIONAL COMPOSITION

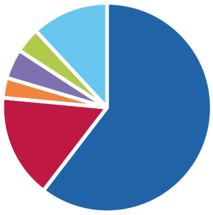

Nitrogen Free Extract	60.4 %
of which Starch	43.5 %
of which Sugars	3.2 %
Crude Protein	16.1 %
Crude Fat	3.1 %
Crude Ash	4.6 %
Crude Fiber	3.9 %
Moisture	11.9 %

ENERGY CONTENT

	MJ/kg	kcal/kg	%
ME Pig	13.2	3 145	
ME Atwater	14.0	3 339	
Energy from proteins	2.7	644	19.3
Energy from lipids	1.2	279	8.4
Energy from NFE	10.1	2 416	72.4

More information on energy calculation: www.safe-lab.com

Analysis End Product

TOTAL PER KG

AMINO ACIDS

Arginine	9 000 mg	Méthionine	2 800 mg
Cystine	2 500 mg	Tryptophane	1 900 mg
Lysine	7 200 mg	Glycine	8 100 mg

FATTY ACIDS

Palmitic acid	5 900 mg
Stearic acid	600 mg
Palmitoleic acid	150 mg
Oleic acid	4 800 mg
LA	15 000 mg
ALA	1 200 mg

MINERALS

	END PRODUCT
Calcium	7 300 mg
Phosphorus	5 500 mg
Sodium	2 500 mg
Potassium	6 000 mg
Magnesium	1 600 mg
Manganese	70 mg
Iron	270 mg
Copper	16 mg
Zinc	55 mg
Chlorine	4 000 mg

VITAMINS

	END PRODUCT
Vitamin A	7 500 IU
Vitamin D3	1 000 IU
Vitamin E	30 IU
Vitamin K3	2.5 mg
Vitamin B1	5.0 mg
Vitamin B2	6.5 mg
Vitamin B3	70 mg
Vitamin B5	10 mg
Vitamin B6	3.0 mg
Vitamin B9	0.35 mg
Vitamin B12	0.010 mg
Biotin	0.080 mg
Choline	1 600 mg

Figure S1. SAFE A04 scientific diet product data sheet (extract).
